# Transfer of Natural Radionuclides in Terrestrial Food Chains—A Review of Investigations in Finland

**DOI:** 10.3390/ijerph182010577

**Published:** 2021-10-09

**Authors:** Susanna Salminen-Paatero, Jussi Paatero

**Affiliations:** 1 Finnish Meteorological Institute, P.O. Box 503, FI-00101 Helsinki, Finland; jussi.paatero@fmi.fi; 2 Department of Chemistry, University of Helsinki, P.O. Box 55, FI-00014 Helsinki, Finland

**Keywords:** natural radionuclides, radioecology, food chain, bioaccumulation, uranium, polonium, radiolead, lichen, reindeer, radionuclide exposure

## Abstract

Transfer of natural radionuclides ^210^Pb, ^210^Po, ^238^U, and ^228,230,232^Th in subarctic food chains has been studied in Finland since the 1960s. The unique food chain lichen-reindeer-man related to Sami people in Finnish Lapland and other food chain options, from berries or mushrooms to man, have been explored and the activity concentrations of natural radionuclides in biological samples determined. The results from Finnish radioecological studies are summarized and differences in bioaccumulation between different radionuclides are discussed. It was found out that, although a substantial amount of activity concentration data exist from the research projects executed in Finland during the last 6 decades, more data, especially from U and Th, in biological environment and humans would be useful, e.g., for modeling purposes and for improved assessment of bioaccumulation and adverse effects (both radiological and chemical) of radionuclides.

## 1. Introduction

Following the atmospheric nuclear tests in the 1950s and early 1960s, several radioecological research projects focusing on the (sub)arctic food-chain lichen-reindeer/caribou-man were initiated in Scandinavia and North America [1,2,3]. Lichen collects deposited radionuclides efficiently and is the main fodder for reindeer during the winter season. The enrichment of radionuclides in this food chain can lead to exceptionally high body burdens among the indigenous Sami and Inuit populations consuming large quantities of the meat and edible organs of reindeer and caribou. This concerns both artificial and natural radionuclides, even though the first research projects dealt with ^137^Cs and other fission product nuclides. However, it was observed that the natural radionuclides ^210^Pb and ^210^Po are enriched in this food chain as well.

^210^Pb is formed in the atmosphere from the radioactive noble gas ^222^Rn, emanating from the Earth’s crust. In total, 99% of the airborne ^222^Rn originates from land and only 1% from the sea [4], and consequently high ^210^Pb concentrations are found in continental air masses. Owing to the long half-life of ^210^Pb (t½ = 22.3 a), its removal from the atmosphere is governed by the wet and dry deposition processes affecting the aerosol particles carrying it rather than radioactive decay. ^210^Pb decays to ^210^Po (t½ = 138.4 d) via the short-lived beta emitter ^210^Bi (t½ = 5.013 d). Being an alpha emitter ^210^Po is highly radiotoxic.

From the radiation protection point of view, less important natural radionuclides are ^238^U, ^226^Ra, ^40^K, and Th isotopes. However, ^238^U is a long-lived (t½ = 4.5 × 10^9^ a) alpha emitter whose chemical toxicity exceeds its radiological toxicity. Although isotope ^238^U is not considered to cause significant radiation exposure, it is still the mother nuclide of a whole uranium decay series, producing isotopes of astatine, bismuth, lead, polonium, protactinium, radium, radon, thallium, and thorium while decaying. ^40^K also has a very long half-life (t½ = 1.28 × 10^9^ a). A human body has a ^40^K whole body content of a few kBqs. The alpha emitter ^226^Ra (t½ = 1600 a) is an important radionuclide considering ingested radiation doses. However, the population in Finland receives exposure to ^226^Ra mainly via uptake from drinking water, and there is hardly any published data about ^226^Ra in terrestrial environment and biota in Finland, except ^226^Ra concentration in cereals [5], animal-based food products [6], and timber [7]. Therefore, ^226^Ra is left outside the scope of this article. Thorium has several isotopes with widely different half-lives. The longest half-life belongs to isotope ^232^Th (t½ = 1.405 × 10^10^ a). Owing to the long half-life, the specific activity of ^232^Th is low and thus its potential threat against human health might be due to its chemical characteristics (heavy metal) rather than radiological hazards.

The main aim of this literature review is to summarize the obtained data concerning natural radionuclides in the food-chains lichen-reindeer-man, mushroom/vegetation-reindeer-man, and mushroom/vegetation-man in Finnish Lapland (northern Finland), and some supporting data is included. A large amount of historical environmental radioactivity data was published only in reports with limited availability. Therefore, it is feasible to collect old and newer research data of natural radionuclides in terrestrial environment in Finland into this single review. A corresponding literature review about transuranium radionuclides in the subarctic food chains in Finland has been published previously [8].

## 2. ^210^Pb and ^210^Po

**Figure 1:**
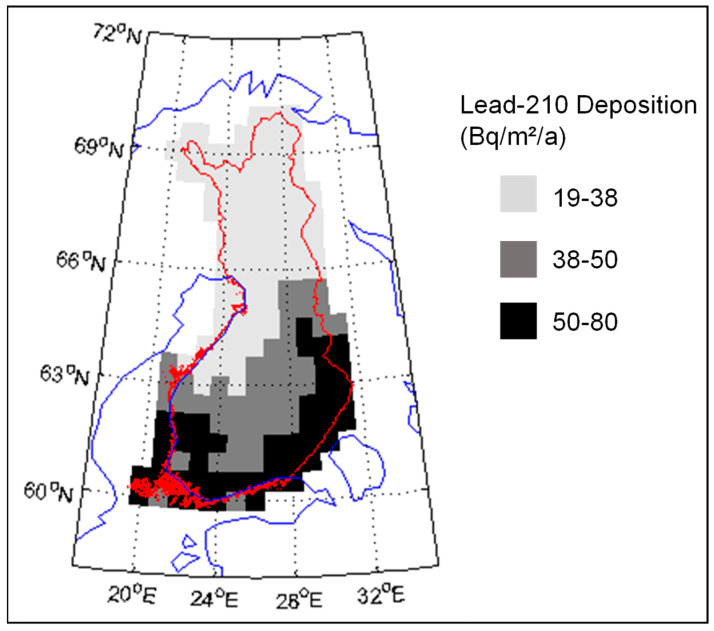
Deposition of ^210^Pb (Bq/m^2^/a) in Finland. Original data is published in [9].

According to Paatero et al. [9], the ^210^Pb deposition in northern Finland is about 20–40 Bq/m^2^/a (Figure 1). The deposition level is lower than in southern Finland, where the maximum deposition is about 80 Bq/m^2^/a. In the atmosphere the ^210^Po:^210^Pb, activity ratio is usually about 5–10%, so the amount of ^210^Po deposition in Finnish Lapland is about 2 Bq/m^2^/a [10]. However, the ^210^Po:^210^Pb activity ratio continues to increase after the deposition.

^210^Po is enriched from soil to plants via root uptake [11,12] and in less extent via foliar uptake. Lichens behave differently than plants as ^210^Po transfers and enriches from air and surface soil to lichens mainly by surface (aerial) uptake [13]. ^210^Pb, a grandmother radionuclide of ^210^Po, has a different chemical and radiochemical behaviour than ^210^Po, although some similarities occur. Just like ^210^Po, ^210^Pb has also been found to accumulate efficiently to lichen, similarly with stable lead [14]. However, as will be discussed next and can be seen from the data in Table 1 and Table 2, there are clear differences between ^210^Po and ^210^Pb in their bioavailability and enrichment in the food chains.

Lichen is the main fodder of reindeer during the winter. In the 1960s, the ^210^Pb activity concentration in lichen (*Cladonia alpestris*) varied between 210 and 380 Bq/kg dry weight (d.w.) in Finnish Lapland and between 190 and 300 Bq/kg d.w. in southern Finland [15]. The ^210^Po:^210^Pb activity ratio in lichen varied from 0.78 to 0.98. The activity concentrations of ^210^Pb and ^210^Po were about a factor of 50 higher in lichen compared to those in other plants that reindeer eat during the summer season, such as birch (*Betula verrucosa/Betula pendula*) leaves. Persson [19] found similar ^210^Pb activity concentration levels in lichen in Sweden. According to his studies, the effective half-life (physical + biological) of ^210^Pb in lichen is 7 ± 2 years. As ^210^Pb and ^210^Po are natural radionuclides, their amount in the environment is very stable in the long-time scale. Due to this, the ^210^Pb content in lichen has not changed during the several decades of monitoring. The activity concentration of ^210^Pb in ground lichen (*Cladonia* sp.) was 250 Bq/kg d.w. in western Lapland in 1980 and 170 Bq/kg d.w. in eastern Lapland in 2004 [16].

**Figure 2:**
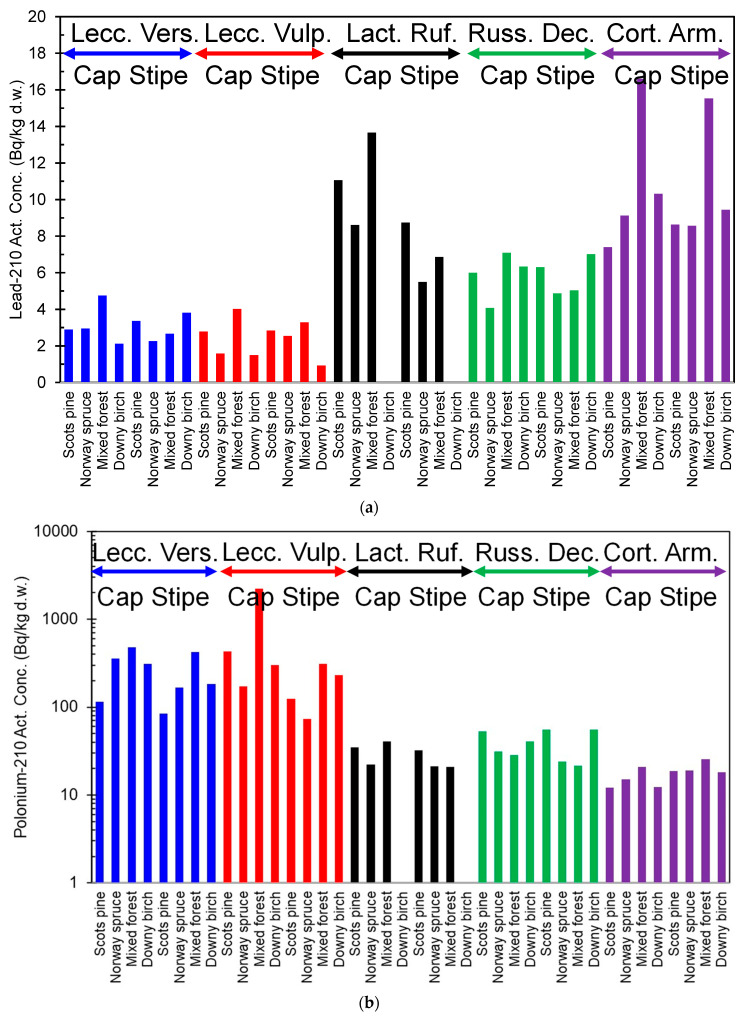
(**a**). ^210^Pb activity content of different mushroom species (blue = *Leccinum Versipelle,* red = *Leccinum Vulpinum*, black = *Lactarius Rufus,* green = *Russula Decolorans*, and purple = *Cortinarius Armillatus*) in northern Finland, from the data in [17]. (**b**). ^210^Po activity content of different mushroom species (blue = *Leccinum Versipelle,* red = *Leccinum Vulpinum,* black = *Lactarius Rufus,* green = *Russula Decolorans*, and purple = *Cortinarius Armillatus*) in northern Finland, from the data in [17].

Reindeer consume mushrooms in late summer and autumn. The ^210^Pb content in brown-yellow boletus (*Suillus luteus*) is rather low, only a few Bq/kg d.w. However, they contain 100–140 Bq/kg d.w. of ^210^Po, which significantly increases the intake of this nuclide into reindeer [16]. The content of ^210^Pb and ^210^Po varies two orders of magnitude from one species to another in mushrooms sampled in northern Finland (Figure 2a,b) [17]. The activity concentration of ^210^Pb ranged from 1.55 (*Leccinum vulpinum*) to 16.2 (*Cortinarius armillatus*) Bq/kg d.w. For ^210^Po, the corresponding range was from 8.98 (*Hygrophorus camarophyllus*) to 2192 (*Leccinum vulpinum*) Bq/kg d.w., respectively. Additionally, the type of forest and forest soil affects the content of ^210^Pb and ^210^Po in mushrooms. For example, the concentration level of radionuclides in the organic-rich litter layer affects the radionuclide content of mushrooms, but a clear connection between soil type and concentrations of ^210^Pb and ^210^Po in mushrooms has not been established yet. Furthermore, mushroom species are suggested to be the most important factor in radionuclide accumulation [17].

**Figure 3:**
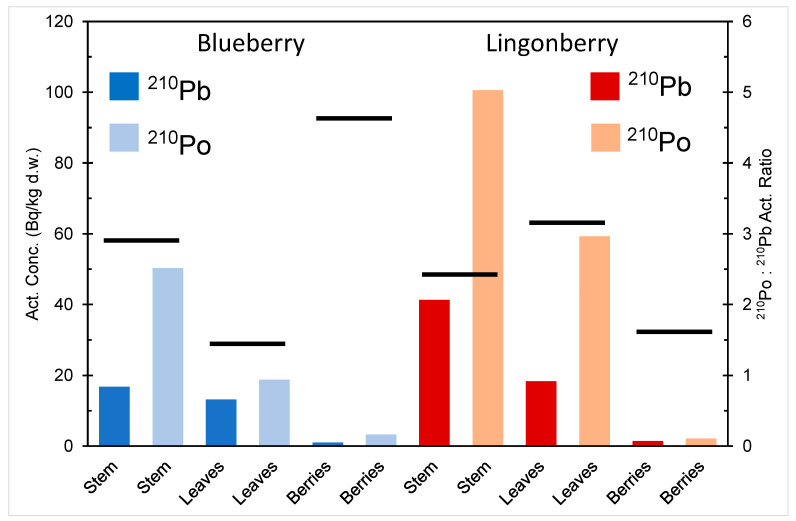
^210^Pb and ^210^Po activity content in different parts of blueberry (*V. myrtillus*) and lingonberry (*V. Vitis-idaea*) in northern Finland, drawn from the data in [17]. The black lines denote the ^210^Po:^210^Pb activity ratios: a vertical scale on the right.

The activity concentration of ^210^Pb and ^210^Po in different parts of blueberry and lingonberry in northern Finland is depicted in Figure 3 [17]. The activity concentration of ^210^Po is higher than that of ^210^Pb in stems, berries, and leaves of both berry species, indicating a higher bioavailability of polonium compared to lead. The berries have much smaller activity content of both radionuclides compared to other parts of the plants. Plants are known to have a selective ability to metal uptake, capacity to store toxic metals in inert parts (e.g., tree stems) and seedlings, and prevention mechanism for blocking accumulation of toxic metals in their reproductive organs, e.g., seeds and berries [20,21,22,23,24].

**Figure 4:**
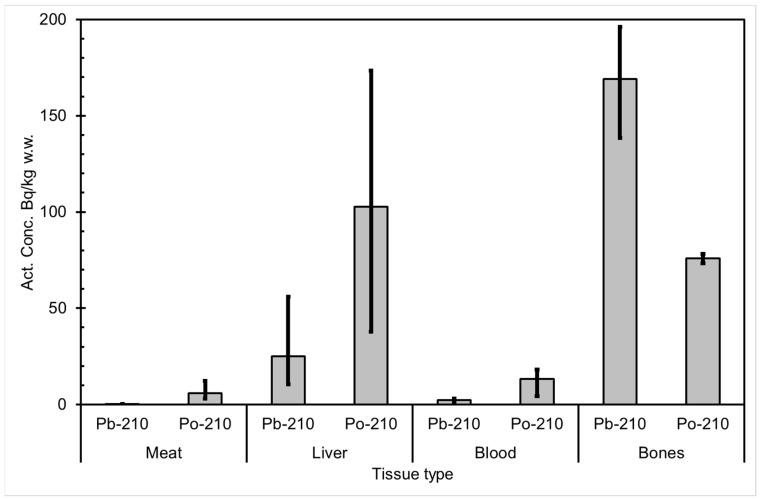
Average ^210^Pb and ^210^Po activity concentrations (Bq/kg wet weight) in reindeer tissues, compiled from data in [15]. The black vertical lines indicate the range of individual values.

**Figure 5:**
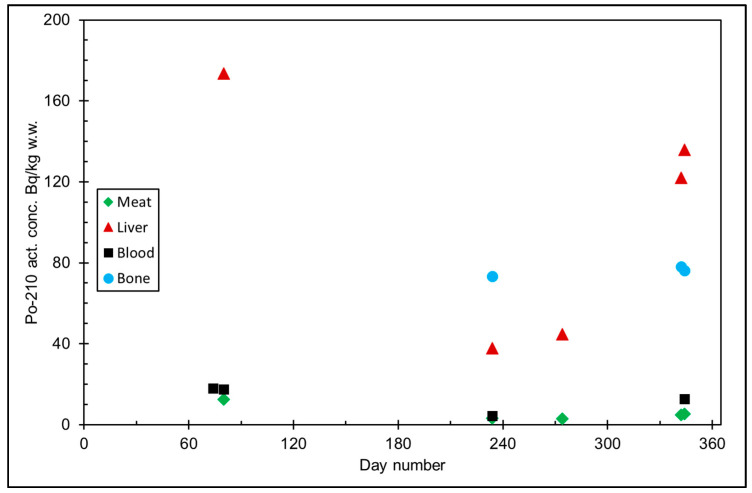
^210^Po activity concentration (Bq/kg w.w. = wet weight) in reindeer tissues, from the data in [15]. Day number refers to Julian day number.

**Figure 6:**
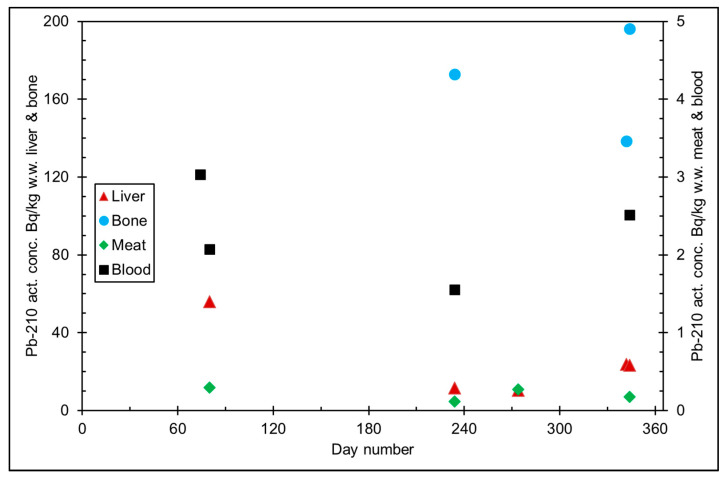
^210^Pb activity concentration (Bq/kg w.w. = wet weight) in reindeer tissues, from the data in [15]. Day number refers to Julian day number.

The seasonal variation of reindeer’s feeding habits are also reflected in the content of ^210^Pb and ^210^Po in various reindeer tissues (Figure 4). The activity concentrations are in their minimum in summer when reindeers have consumed vascular plants, birch leaves, etc. (Figure 5 and Figure 6). The activity concentrations are higher in autumn and winter after the consumption of first mushroom and, later, lichen [15].

According to results by Solatie et al. [16], ^210^Pb mainly enriches reindeer bones after entering to reindeer body. There was a 10-fold concentration of ^210^Pb in the reindeer bones compared to muscles. In the same study, the concentration of ^210^Po was two times higher in bones than in muscles, at most. Therefore, relative enrichment of ^210^Po to bones was not as high as of ^210^Pb in investigated samples. Alongside bones, both ^210^Pb and especially ^210^Po enrich the liver and kidney in reindeer. The same preference for enrichment of ^210^Pb was also observed with aribous in Canada [13]. In past centuries when reindeer was the foundation of diet among reindeer herders, consumption of inner organs and bone marrow of reindeer was also considerable; therefore, it has had a significant effect on intake of both ^210^Pb and ^210^Po, increasing the internal radiation dose in the Lapland region.

Enrichment of ^210^Pb and ^210^Po from diet to humans is roughly similar to reindeer, since both radionuclides accumulate to human bone, liver, and kidney. Next, some differences between the two regions (Lapland and southern Finland) with different diets in the past, in the concentrations of ^210^Pb and ^210^Po in human organs, are discussed. A related review about possible increased cancer risk among Sami people due to anthropogenic radionuclides from nuclear weapons tests, diet, and life habits has been recently published by Soininen and Mussalo-Rauhamaa [25].

Kauranen and Miettinen [15] estimated that the intake of ^210^Po via ingestion within the reindeer herding population, 2.6 Bq/d, was 20 times higher than in the case of the general population, having an average western diet of 0.12 Bq/d. The corresponding intake of ^210^Pb was three times higher, 0.32 Bq/d, within the reindeer herding population, than in the average western diet of 0.12 Bq/d. The high intake of ^210^Po was mainly due to ^210^Po in reindeer meat and liver. In recent decades, the difference in diet habits between reindeer herders vs. the average population has probably decreased but not entirely disappeared. The high ^210^Po content in diets is also reflected in the body content of ^210^Po within the reindeer herding population. The ^210^Po activity concentration in the blood of reindeer herders was almost 20 times higher than within the population of Helsinki. The difference in the case of ^210^Pb was almost threefold. Correspondingly, the activity concentration of ^210^Po in human placenta in Inari and Utsjoki was over 12 times higher than within the population of Helsinki. In the case of teeth, the difference was only about a factor of two; the same as with ^210^Pb. It is clear that ^210^Po is not accumulated into teeth but is formed in situ from the decay of ^210^Pb.

In a later study by Mussalo-Rauhamaa et al. [18], a set of tissue samples of five autopsy cases from Inari and Utsjoki region, deceased between 1977 and 1979, were analyzed. It was found that the ^210^Pb activity concentration in human liver ranged from 0.13 to 1.0 Bq/kg wet weight (w.w.) with an average value of 0.48 Bq/kg w.w. In autopsy cases from southern Finland, an average ^210^Pb activity concentration of 0.27 Bq/kg w.w. was obtained. ^210^Po activity concentration in human liver in Lapland ranged from 0.40 to 8.0 Bq/kg w.w., with an average of 3.2 Bq/kg w.w. In autopsy cases from southern Finland, an average ^210^Po activity concentration of 0.57 Bq/kg w.w. was obtained. Thus, the activity concentration was two times higher in the residents of Lapland compared to those in southern Finland in the case of ^210^Pb. In the case of ^210^Po, the average activity concentration was over five times higher in the residents of Lapland compared to those in southern Finland. In a recent study, Muikku et al. [26] reported that the ^210^Po content in urine was three times higher in reindeer herders than the control group representing the average population. However, in the case of ^210^Pb, there was no difference.

The work of Holm and Persson [27] indicates clearly how ^210^Po dominates the total alpha activity of lichen (Table 3). The activity concentration of ^210^Po in 1972 was 50 times higher than the infamous plutonium isotopes from the atmospheric nuclear tests. In addition, the deposition of ^210^Pb has recurred every year after the ice age and will recur in the future too, contrary to plutonium deposition, which was in a longer timescale and was practically a single event. Furthermore, the half-lives of artificial radionuclides ^238^Pu (t½ = 87.7 a), ^244^Cm (t½= 18.1 a), and ^242^Cm (t½ = 163 d) are so short that they have decayed more or less significantly since their introduction to the environment.

After 1972, there have been several other nuclear events releasing artificial radionuclides to the environment, most importantly the nuclear reactor accidents of Chernobyl in 1986 and Fukushima in 2011. This kind of accidental release of radionuclides creates local and regional elevated radiation exposure. The radionuclide(s) in question, as well as the released activity level, environmental conditions, and countermeasures after the accident, etc., determine how high fractions of the received radiation dose are from natural radionuclides and from accidentally released (often artificial) radionuclides. The early situation after a nuclear accident, where artificial radionuclides often dominate the natural radionuclides as an exposure source, may change during time, when shorter-lived artificial radionuclides have been decaying and both spontaneous environmental processes, as well as remediation actions, have occurred.

The consumption of mushrooms and berries in Finland gives an example about changing radiation dose contributions due to new radioactivity sources. Deposition of ^137^Cs from the Chernobyl accident was uneven and parts of southern and central Finland received high deposition of ^137^Cs, whereas Finnish Lapland remained almost untouched. Therefore, ^210^Po and ^210^Pb in mushrooms and berries are still the main factors in their resulting ingested radiation dose in Lapland, while in southern Finland, Chernobyl-originated ^137^Cs has been the most important radionuclide, causing radiation doses from consumption of mushrooms and berries after 1986 [17].

In addition to radioecological studies, determination of ^210^Pb and ^210^Po from peat and sediment samples has been widely utilized in age dating purposes in Finland [28,29,30,31,32,33,34,35]. Applications of ^210^Po and ^210^Pb age dating have been found in, e.g., geology, environmental pollution studies, ecology, biogeochemistry, and climate change research.

## 3. Uranium Isotopes

There is not much published data about uranium concentrations in the terrestrial, above-ground environment in Finland (Table 4). The reasons for not studying or publishing uranium concentrations can be only speculated; maybe the concentration in the air, deposition, soil, and biota has been considered low, in respect to biological enrichment and health risks. Extensive investigations into U concentration in the Finnish geological environment have been conducted, but they have focused on studying conditions in the bedrock during and after the last ice age and characterizing chemical properties of the bedrock for nuclear waste disposal. Additionally, natural U deposits (natural analogue sites) and U-rich rock formations have been important research topics of radionuclide migration in Finland. In these geochemical studies, determination of U and Th concentrations, oxidation states of U, as well as sample age determination via U and Th isotope ratios, have been utilized as research methods [36,37,38,39].

The majority of ingested uranium in Finland, and possibly following health effects from uranium exposure, is due to consumption of drinking water containing substantial amounts of ^238^U and its daughters. Since this review focuses on exposure and enrichment route via ingestion of food, natural radionuclides in Finnish drinking waters are excluded from this overview, as that topic should earn its own literature review. Health effects from uranium decay series radionuclides due to drinking water consumption in Finland have been published by, e.g., Kahlos and Asikainen [42], Auvinen et al. [43], Kurttio et al. [44], Vesterbacka et al. [45], Vesterbacka [46], Kurttio et al. [47], and Turtiainen et al. [48]. However, enrichment of uranium from soil to food products and further to humans has been proved, e.g., in Germany [49] and Canada [13].

Yliruokanen [40] investigated the effect of uranium-rich bedrock on natural radionuclide concentrations of above-growing plants. The plant samples were collected in different locations in southern Finland in 1972 [50]. The published U concentrations in the plant ash varied from one plant group to another. The reported values were converted to a concentration of U in dried plants, shown in Table 4. Mosses (*Cetraria islandica, Pleurozium schreberi, Sphagnum* sp., *Dicranum* sp., *Polytrichum commune, Hylocomium splendens,* and *Rhacomitrium microcarpon*) had the highest U concentrations, 0.16–47 mg/kg d.w. The moss sample with the highest U concentration value was growing within proximity of U-rich pegmatite formation. Other plant species had a 10-fold variation in U concentrations: lichens (*Cladonia alpestris*, *CI. arbuscula*) had U concentrations of 0.06–0.57 mg/kg d.w., blueberry (*Vaccinium myrtillus*) had 0.05 mg/kg d.w., lingonberry (*Vaccinium vitis-idaéa*) had 0.05 mg/kg d.w., silver birch (*Betula alba*) had 0.04–0.12 mg/kg d.w., and conifers (*Pinus sylverstris* and *Picea abies*) had 0.03–0.51 mg/kg d.w. [40].

Roivainen et al. [41] studied four different plant species, in respect to U accumulation from soil to plants, using concentration ratios. The investigated plant species were May lily (*Maianthemum bifolium*), narrow buckler fern (*Dryopteris carthusiana*), rowan (*Sorbus aucuparia*), and Norway spruce (*Picea abies*). They found that all four plant species behaved quite similarly, the mean U concentrations being 0.01–0.02 mg/kg d.w. (0.12–0.25 in Bq/kg) in leaves or needles and 0.09–0.17 mg/kg d.w. (1.1–2.1 in Bq/kg) in coarse roots. In all species, the concentration ratio between the plant and soil was higher in roots than in leaves/needles.

Indeed, high accumulation of U in plant roots and preference for remaining in the root system instead of above-ground plant parts has been observed in many studies, both in water and soil cultivation and with naturally growing plants [51,52,53,54,55].

One study came to the opposite conclusion; the green parts of the common nettle (*Urtica dioica*) contained more U than roots in the vicinity of a phosphogypsum stockpile [56]. Furthermore, U concentration in the plants did not decrease according to increasing distance from the stockpile. A probable reason for these observations was suspected to be due to aerial uptake of deposition by the plants. In any case, soil properties, plant species, plant age, U concentration of soil, type of root system, U speciation, etc., affect the accumulation of U in plants. Another contradictory study utilized a variety of plants and soil layers O and C beneath them, collected from a wide sampling area across northern Europe, including Finland [57]. Plant samples blueberry (*Vaccinium myrtillus*), cowberry/lingonberry (*Vaccinium vitis-idaea*), crowberry (*Empetrum nigrum*), birch (*Betula pubescens*), willow (*Salix spp*.), pine (*Pinus sylvestris*), and spruce (*Picea Abies*) contained extremely low concentration of U, and plant/soil ratio could not be determined due to this. Moss samples (*Hylocomium splendens* and *Pleurozium schreberi*) were an exception, since they contained low but detectable amounts of U: 0.029 mg/kg d.w. was the median value of 140 samples. This study concluded that U remains in the C layer, instead of accumulating to plants via root uptake.

Uranium mostly enriches the kidneys, but also the skeleton and, to lesser extent, other internal organs after entering to human body. The predominant exposure source of U for humans in Finland is drinking water [44]. However, studies on increased cancer risk due to U and other natural radionuclides in Finnish drinking waters, including drilled wells in the U-rich bedrock, have not found clear correlation between cancer incidents and natural radionuclide content in drinking water [43,47].

## 4. Thorium Isotopes

Compared to ^238^U, there is even less published data about Th concentrations in the Finnish environment. Th, being at valence state 4+ in the environment, is less soluble and mobile than U, which is hexavalent in aerobic conditions and tetravalent in anoxic conditions in the environment. Thus, Th is less available for biota as well. Concentration of Th isotopes in the environment is lower than that of ^238^U. This can be also seen from the literature values of Reference [58] (Table 5), where activity concentrations of Th isotopes are shown to be much lower than the corresponding values of ^238^U. Higher concentrations of Th, equal to U, were found from plant samples growing close to or on a U-rich formation [40]. Transfer of Th from soil to food products has been observed in several international studies, e.g., [59,60,61,62], as well as accumulation of Th isotopes to humans from ingestion or inhalation, as in References [63,64,65].

Th resembles U in transferring from water (in hydroponic cultivation tests) or soil to plants via root uptake, because a major part of Th remains in the roots and only a minor part relocates to other parts of the plants [54]. Th was also found out to be bioaccumulated into wheat seedlings from water and soil in a phytoremediation study [66].

Yliruokanen [40] found out that ^232^Th concentration was low in plant ash, regardless of the plant species, being 0.24–4.1 Bq/kg d.w. (0.06–1.0 mg/kg d.w.) in lichens (*Cladonia alpestris*, *CI. arbuscula*), mosses (*Cetraria islandica, Pleurozium schreberi, Sphagnum* sp., *Dicranum* sp., *Polytrichum commune, Hylocomium splendens, Rhacomitrium microcarpon*), heather (*Calluna vulgaris*), blueberry (*Vaccinium myrtillus*), lingonberry (*Vaccinium vitis-idaéa*), and conifers, while it was below the detection limit in deciduous trees. The sampled plants were growing in areas with U-rich bedrock. Additionally, the concentration of ^232^Th in the investigated plants was stable considering varying mineralogical compositions and radionuclide levels in the bedrock below among the sampling sites.

According to Muuronen [58], three thorium isotopes were present in lichen samples of Lapland, namely ^232^Th (t½ = 1.405 × 10^10^ a), ^230^Th (t½ = 75400 a), and ^228^Th (t½ = 1.913 a). The activity concentration ranges varied from 0.076 to 0.47 Bq/kg d.w., from 0.063 to 0.19 Bq/kg d.w., and from 0.12 to 0.57 Bq/kg d.w. for ^232^Th, ^230^Th, and ^228^Th, respectively. In central Sweden, the corresponding activity concentrations were 0.06, 0.06, and 0.13 Bq/kg d.w., respectively; in other words, they were of the same order of magnitude as in northern Finland [27].

Muuronen [58] determined the same Th isotopes from reindeer bone samples as well. The activity concentration of ^228^Th, 2.4–23 Bq/kg d.w., was significantly higher in the reindeer bone samples compared to that of ^232^Th, 0.011–0.096 Bq/kg d.w., and ^230^Th, 0.012–0.11 Bq/kg d.w. This was due to presence of ^228^Ra, the mother nuclide of ^228^Th, in the reindeer bones. Young reindeers had much lower ^228^Th concentration in their bones compared to old reindeer individuals, who had a longer history of consuming lichen and other plants containing natural radionuclides. Therefore, there is no isotopic fractionation of Th isotopes in bioaccumulation to reindeer, based on these results.

Th enriches human skeletons just like U does. However, it was observed in the study by Larivière et al. [65] that, while accumulation of U was age-dependent in bone samples and it correlated with calcium turnover rate in the bones, Th accumulation in the bones was not age-dependent. The activity concentrations of Th isotopes in human bones in Finland have been determined to be 0.002–0.003 Bq/kg w.w. for ^232^Th, 0.004–0.005 Bq/kg w.w. for ^230^Th, and 0.010–0.013 Bq/kg w.w. for ^228^Th [58].

## 5. Radiation Dose Due to ^210^Pb and ^210^Po from Consumption of Reindeer Meat

Solatie et al. [16] calculated that a reindeer herder receives an effective radiation dose of 0.7 mSv/a when he/she consumes 200 g of reindeer meat daily, and the measured activity concentration of ^210^Po in the reindeer meat is 9 Bq/kg. The radiation dose increment from reindeer meat ingestion is 19% of the total annual average radiation dose from all sources in Finland. For comparison, effective annual radiation dose from inhalation of ^210^Pb in Finnish Lapland has been estimated to be 7 µSv/a, so the dose from inhaled ^210^Pb would be only 1% of the dose from ^210^Po via daily consumption of reindeer meat [67]. As previously presented, the radiation dose from other natural radionuclides is negligible compared to that from ^210^Pb and ^210^Po. However, other natural sources of radiation exposure, such as indoor radon, cosmic radiation, and gamma radiation from the ground, are the main factors contributing to the annual dose.

Thomas and Gates [13] calculated radiation doses from ingesting natural radionuclides in the case of the food chain lichen-caribou-man in northern Canada. They assessed effective annual doses from consuming meat, liver, and kidney separately and together. In their most similar case to the aforementioned calculation in [16], 100 g of caribou meat was consumed daily, and the resulting effective dose from ^210^Po was 0.61 mSv/a. The estimated radiation doses due to ingestion of reindeer meat in northern Finland and caribou meat in northern Canada are similar. Furthermore, consuming 100 g of caribou meat daily, one caribou liver per year, and 10 caribou kidneys per year would produce in total an effective radiation dose of 1.2 mSv/a only from ^210^Po [13], but other natural radionuclides have their own inputs to the received radiation dose as well.

## 6. Summary of Highlights in Natural Radionuclide Studies in Terrestrial Environment in Finland in Recent Decades

**Figure 7:**
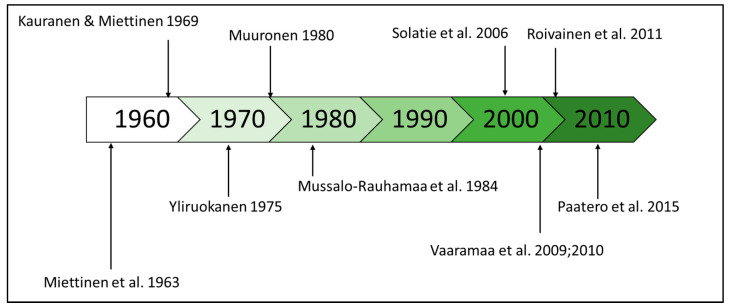
Timeline of the most significant steps in natural radionuclide studies in terrestrial environment in respect to food chains in Finland.

Highlights of the articles cited in this review are presented in the timeline in Figure 7. Interest in natural radionuclides as a source of radiation exposure in Finland is constantly increasing among decision-makers, research funding bodies, and research institutes, mainly due to NORM- (naturally occurring radioactive materials) related risks associated with existing and planned mines. New research openings will probably bring substantial amounts of new data about natural radionuclides in the Finnish environment, which is long-awaited and necessary as there are gaps in the timeline and only a few literature sources are available from the past six decades.

## 7. Conclusions

Considerable amounts of data concerning the transfer of natural radionuclides, especially ^210^Pb and ^210^Po, along with the subarctic food-chain lichen-reindeer-man have been gathered during the last 5–6 decades. Still, concentrations and bioaccumulation of natural radionuclides in terrestrial food chains are not fully known, and it would be useful to have more data of radionuclide concentrations in the biological samples in Finland. For example, U and Th have been much less investigated compared to Pb and Po, both in the environment and in humans. More numerous data would enable more reliable and accurate evaluation of radionuclide transfer and adverse effects due to radionuclide bioaccumulation.

Compared to studies performed in other subarctic regions, natural radionuclide concentrations in the Finnish terrestrial environment and biota are at the same level, although high variations exist in activity concentration values of a radionuclide. When comparing internal ingested radiation doses from foods among a wider group of populations, the highest concentration values of natural radionuclides in edible plants, mushrooms, and reindeer need to be notified due to the common practice of collecting natural food items and due to the specific reindeer herding tradition in Finland.

Differences in concentration levels and bioaccumulation of ^210^Pb and ^210^Po between two population groups, Sami people in Lapland and people in southern Finland, are clear, since they have had different diets until recent decades. It has been concluded that ^210^Po and ^210^Pb are the most important radionuclides in Lapland and ^137^Cs from the Chernobyl accident is the main contributor in southern Finland, while considering the radiation dose from consuming mushroom and berries in recent decades [17]. Food chain studies are constantly important in the evaluation of human radiation exposure both locally and globally as there are radioecologically exceptional environments for living, cultivating, hunting, and collecting and regional diets containing established and specific food chains. Furthermore, the level of radioactive contamination and bioavailable radionuclides in environments, both natural and artificial, will probably increase in the future. Forthcoming sources of environmental radioactivity can include accidental and controlled releases from nuclear power plants, nuclear fuel reprocessing facilities, and nuclear weapons testing. In addition, the operation of uranium mines and other mines releasing NORM radionuclides into the environment is likely to increase in the future.

Various radioecological and radiation protection models have been developed to assess the transfer of radionuclides along food chains and radiation doses due to ingestion. It has been pointed out by Thomas and Gates [13] that these models do not necessarily take into account chemical toxicity of the radionuclides, in addition to radiochemical and radiobiological effects. Radionuclides are often chemical analogues to stable elements in humans, for example, Po and S are chemical analogues and, therefore, Po can replace S in proteins. Not all of these special features of radionuclides in biological processes are included in the current radiation exposure and radionuclide transfer models; therefore, models need further development for obtaining improved views about the effects of biological enrichment of radionuclides in the environment.

## Figures and Tables

**Table 1:** Concentrations of ^210^Pb in Finnish environmental and biological samples. d.w. = dry weight, w.w. = wet weight. Sampling year is in parentheses.

Sample Type	A ^210^Pb (Bq/kg d.w.)	A ^210^Pb (Bq/kg w.w.)	Reference
Lichen (1961–1967)	190–380		[15]
Lichen (1980)	250		[16]
Lichen (2004)	170		[16]
Tree leaves (1964–1965)	11–42		[15]
Tree leaves (1989)	20		[16]
Mushroom (2004)	3		[16]
Mushrooms (2007)	1.6–16		[17]
Berries (2004)	2		[16]
Berries (2006–2007)		0.09–0.45	[17]
Reindeer blood (1965–1967)		1.5–3	[15]
Reindeer meat (1965–1967)		0.12–0.30	[15]
Reindeer muscle (1984–2004)	1.2–4.8		[16]
Reindeer bones (1964–1966)		138–196	[15]
Reindeer bones (1988–2004)	38–210		[16]
Reindeer kidney (1988–1989)	62–85		[16]
Reindeer liver (1964–1967)		10–56	[15]
Reindeer liver (2004)	150		[16]
Human blood, reindeer herder (1966)		0.23–0.27	[15]
Human blood, southern Finland (1966–1967)		0.1	[15]
Placenta, Lapland (1966)		0.02–0.12	[15]
Placenta, southern Finland (1966)		0.01–0.05	[15]
Human kidney, southern Finland (~1966)		0.12–0.24	[15]
Human teeth, Lapland (1966–1967)	2.4–11		[15]
Human teeth, southern Finland (1966–1967)	1.4–2.6		[15]
Human shinbone/tibia, southern Finland (~1966)		1.0–1.5	[15]
Human ribs, Lapland (1977)		0.85 and 1.5	[18]
Human liver, Lapland (1977–1979)		0.13–1.0 (average 0.48)	[18]
Human liver, southern Finland (~1966)		0.08–0.11	[15]
Human liver, southern Finland (1976–1979)		0.27 (average)	[18]

**Table 2:** Concentration of ^210^Po in Finnish environmental and biological samples. Sampling year is in parentheses.

Sample Type	A ^210^Po (Bq/kg d.w.)	A ^210^Po (Bq/kg w.w.)	Reference
Lichen (1961–1967)	170–350		[15]
Tree leaves (1964–1965)	3–12		[15]
Tree leaves (1989)	19		[16]
Mushroom (2004)	140		[16]
Mushrooms (2007)	9–2190		[17]
Berries (2004)	2.2		[16]
Berries (2006–2007)		0.3–1.0	[17]
Reindeer blood (1965–1967)		4–18	[15]
Reindeer meat (1964–1967)		3–12	[15]
Reindeer muscle (1984–2004)	23–66		[16]
Reindeer bones (1964–1966)		73–78	[15]
Reindeer bones (1988–2004)	12–71		[16]
Reindeer kidney (1988–1989)	120–160		[16]
Reindeer liver (1964–1967)		38–174	[15]
Reindeer liver (2004)	470		[16]
Human blood, reindeer herder (1966)		0.24–0.57	[15]
Human blood, southern Finland (1966–1967)		0.03	[15]
Placenta, Lapland (1966)		0.63–2.8	[15]
Placenta, southern Finland (1966)		0.05–0.16	[15]
Human kidney, southern Finland (~1966)		0.34–0.95	[15]
Human teeth, Lapland (1966–1967)	2.3–10		[15]
Human teeth, southern Finland (1966–1967)	1.4–2.5		[15]
Human shinbone/tibia, southern Finland (~1966)		0.95–1.3	[15]
Human ribs, Lapland (1977)		0.8 and 1.0	[18]
Human liver, Lapland (1977–1979)		0.4–8.0 (average 3.2)	[18]
Human liver, southern Finland (~1966)		0.34–0.83	[15]
Human liver, southern Finland (1976–1979)		0.57 (average)	[18]

**Table 3:** Alpha-emitting radionuclides in lichen in Sweden in 1972 (original data from Holm and Persson [27]) and relative activities compared to ^238^U.

Radionuclide	Activity Concentration (Bq/kg d.w.)	Relative Activity (Act. Conc. ^238^U = 1)
^210^Po	260	1300
^234^U	0.2	1
^235^U	0.007	0.035
^238^U	0.2	1
^228^Th	0.13	0.65
^230^Th	0.06	0.3
^232^Th	0.06	0.3
^238^Pu	0.23	1.15
^239+240^Pu	5.2	26
^241^Am	1.1	5.5
^237^Np	0.01	0.05
^242^Cm	0.0002	0.001
^244^Cm	0.002	0.01

**Table 4:** Uranium concentration in Finnish environmental samples.

Sample Type	^238^U-conc. (mg/kg d.w.)	^238^U-conc. (Bq/kg d.w.)	Reference
Lichens Mosses Shrubs Trees (deciduous and conifers)	0.06–0.57 0.16–47 0.05 0.03–0.51	0.75–7.1 2.0–584 0.62 0.37–6.3	[40]
May lily, narrow buckler fern, rowan, Norway spruce	0.01–0.02 (leaves and needles) 0.09–0.17 (coarse roots)	0.12–0.25 1.1–2.1	[41]

**Table 5:** Concentrations of Th isotopes in the Finnish environmental and biological samples.

Sample Type	Act. conc. ^232^Th (Bq/kg)	Conc. ^232^Th (mg/kg)		Reference
Lichens, mosses, dwarf shrubs, conifers	0.24–4.1 (d.w.)	0.06–1.0 (d.w.)		[40]
	Act. conc. ^232^Th (Bq/kg)	Act. conc. ^230^Th (Bq/kg)	Act. conc. ^228^Th (Bq/kg)	
Lichen	0.076–0.47 (d.w.)	0.063–0.19 (d.w.)	0.12–0.57 (d.w.)	[58]
Reindeer bone	0.011–0.096 (d.w.)	0.012–0.11 (d.w.)	2.4–23 (d.w.)	[58]
Human bone	0.002–0.003 (w.w.)	0.004–0.005 (w.w.)	0.010–0.013 (w.w.)	[58]

## Data Availability

Not applicable.
